# Roads less traveled: understanding the “why” in simulation as an integrated continuing professional development activity

**DOI:** 10.1186/s41077-019-0111-z

**Published:** 2019-11-11

**Authors:** Walter Tavares

**Affiliations:** 1 The Wilson Centre, University Health Network, 200 Elizabeth St. 1Es-565, Toronto, Ontario M5G 2C4 Canada; 2 Post MD Education, Faculty of Medicine, Univeristy of Toronto, Toronto, Ontario Canada; 3 The Regional Municipality of York, Community and Health Services, Newmarket, Ontario Canada

**Keywords:** Continuing professional development, Continuing medical education, Simulation, Motivation

## Abstract

**Background:**

The simulation community has experienced significant advances, strengthening the case for the use of simulation in medical education toward improving patient outcomes. However, an underlying assumption particularly regarding utilization of simulation by those who are in practice, is that simulation will be selected as a continuing professional development (CPD) strategy. Exploring reasons for choices of educational formats, particularly regarding simulation, is poorly integrated into CPD research.

**Discussion:**

Despite significant advances the scientific simulation community has been slow to produce evidence regarding why practitioners may be reserved in engaging in simulation or not. Using examples from related education contexts the author attempts to bridge simulation science, CPD and less commonly used theoretical frameworks to address this issue. The author argues that theoretical perspectives that recognize the use of simulation for CPD as a socio-personal process and/or a personal or group issue (e.g., theories of intelligence, self-determination theory, theory of planned behavior, social identity theory) and that are conceptually distinct from educational mechanisms/ provision are necessary to advance simulation use in CPD contexts.

**Conclusion:**

Given the close relationship practicing clinicians have to patient outcomes a new imperative may be to focus on the theoretical and practical links informing simulation use for CPD at the level of the individual and individual-among-professional groups. The simulation community may therefore need to engage in research that attempts to further uncover and address underlying issues of “why” clinicians integrate simulation as CPD activities or not.

Finding ways to optimize clinical outcomes remains a priority in healthcare and is a call the simulation community has enthusiastically taken up and continues to refine [1]. Ziv et al. (2003) wrote that “the proper and *careful development of simulation-based* medical education is an ethical imperative” (emphasis added) and one that might serve to reduce the tensions associated with the “unreflective use of patients… as training tools for clinicians.” [2] Consequently, the healthcare simulation community obliged with rapid growth in scholarly activity, technological developments, faculty development strategies, professional societies, dedicated fellowships, and international conferences. Recent systematic reviews now highlight what the community can claim as evidence and indicate where there is still work to be done [3–11]. In trainee contexts, the arguments in favor of simulation are now strong and familiar, and simulation may now be as integrated or at least argued to be as relevant as the archetypical textbook, maybe more so. However, once individuals have transitioned from training into independent practice, it appears that simulation becomes comparatively less integrated as a continuing professional development (CPD) or educational strategy, even with and especially without professional obligations to maintain practice abilities and meet regulatory requirements or other potential drivers. It is not entirely clear why and exploration of these issues tend to be limited, or “roads less travelled”. Given the close relationship practicing clinicians have with clinical outcomes, understanding why simulation may be an underutilized CPD strategy may now constitute a renewed ethical imperative.

As evidence mounts shedding light on how experience alone may not be a suitable surrogate for expertise and how preparing individuals for initial autonomy may not adequately prepare them to respond to changes once in practice [12, 13], broadening our understanding of the issues surrounding the use of simulation for CPD may be necessary. Contemporary healthcare simulation provides many affordances for CPD, but the choices one makes in enacting simulation, or any other strategies, likely involve a kind of “relationship” between one’s identified needs and what might serve as the best solutions. That is, cognitive and social views shaped by interactions or experiences may influence the way an individual enacts their CPD. Consider as an example, the CPD work of Gawande [14], whose 2012 article in *The New Yorker* described the complexity associated with surgical work as well as the expertise that evolves over time with exposure to cases. He described how his performance improved over time when measured against national surgical benchmarks but that this learning curve eventually plateaued. In an attempt to improve further he selected coaching over simulation as his CPD strategy—an educational process he described as including observation, judgment, reflection and guidance. While this proved to be effective, one could ask, why was *simulation* not selected as the CPD strategy? Affordances associated with simulation are well known including similar opportunities for observation, judgment, reflection, and guidance [15, 16]. Further, simulation affords, for example, access to clinical events that are not readily available or achieved in clinical settings but are equally important to clinical outcomes and where repeated practice and productive failures are beneficial without compromising health outcomes [3, 10, 17]. While this example highlights a surgical context, it is likely that these issues exist to some degree and in similar ways across specialities and the health professions. While studies have focused on the *role* of simulation for qualified clinicians [18, 19], we may instead or in addition require a focused program of research exploring the underlying individual, professional and social experiences and interactions—or relationships—practicing clinicians have with simulation that may foster or antagonize its use for CPD.

Despite significant advances in educational simulation, the scientific simulation community has been slow to produce evidence for why practitioners appear to be reserved in *selecting* simulation as a CPD activity. While some have discussed how simulation has been underutilized in CPD [20, 21], the focus in the simulation community remains largely at the level of utility and educational mechanisms. These have included, for example, the effects of developmental strategies [22, 23], comparing effectiveness of simulation designs [24], promoting effective simulations [25–29], links to maintenance of certification [30, 31], consideration of simulation to assess performance or gaps [32], improving quality of systems of care [33, 34], clinician perspectives on changes to knowledge skills and attitudes [33, 35], and simulation uses in CPD [36, 37]. That is, simulation research for CPD does not necessarily or simultaneously provide evidence or input into issues related to its adoption at the level of the individual or professional environment.

When researchers do focus on why simulation may be under-utilized for CPD a number of well-grounded hypotheses have been proposed. These include inertia, habit, absence of accountability, resistance to spotlighting clinician abilities, perceptions of psychological or social safety, evaluation apprehension, perceived limited links to actual clinical outcomes and an ineffective or untrustworthy simulation faculty base [15, 28, 38]. That said, evidence related to these or other claims is limited and solutions are even scarcer. As such, when considering lessons for the use of simulation as a CPD strategy, McGaghie et al., 2009 argued “policies that inform [practicing clinician] performance and govern the privilege of practice not only need to endorse the effective use of simulation technology, but also *tackle sources of cultural resistance to its adoption*” (emphasis added) [38]. Attempting to acknowledge and ultimately overcome individual or system/cultural level barriers is not new [39, 40]. The next step is to expand the work, to test hypotheses, broaden understanding and ask how can simulation in CPD achieve the same level of “hard-wired” integration as it has in trainee contexts [41] or as some have argued coaching has for sports or music [42].

Given the potential affordances offered by simulation-based activities emphasizing “why” practicing clinicians engage in simulation (or not) will likely require the scientific simulation community to complement typically used educational theories (e.g., experiential learning, mastery learning, reflective practice, deliberate practice) with theories emphasizing individual experiences within community and social contexts. Doing so would shift the unit of analysis to recognize simulation in a CPD context as a socio-personal process and a personal or group issue that is conceptually distinct from an educational mechanisms or provision [43]. I argue that the use of multiple and diverse theoretical approaches, either by using theory to inform or by contributing to the development or refinement of theory, will provide new lenses to explore, understand and explain the integration of simulation in CPD contexts. This may help to account for individual practicing clinician experiences with their social influences, realities, beliefs and knowledge.

Consider two examples that highlight the value of exploring different professional contexts when attempting to optimize education strategies. First, Watling et al. [44] highlighted how medical and post-graduate medical trainees differ from those in music. This author group revelaed that medical trainees prefer learning by doing mainly in clinical contexts, strive for competence, and value teacher expertise. By contrast, music trainees value learning through one-on-one instruction and individual practice mainly in practice contexts, strive for ever-better performance, and seek teachers with exceptional instructional skill. Second, Mutaabdzic et al. [45] explored assumptions related to the use of coaching by qualified surgeons. They discovered that the integration of coaching is likely influenced by a number of *individual and cultural issues* including for example, perception of fit, limited motivations by some to improve technically, threats to image (e.g., concerns of portraying incompetence), credibility, autonomy and authority. In considering these findings, the authors concluded that “It might be considered ironic that a surgeons’ culturally embedded value of *performing competence* may be the very thing that prevents further development of competence …. [and that] surgical culture may also limit the format of CPD that surgeons pursue” [45]. The insights gained from these two studies illustrate the benefits of shifting attention away from a sole focus on educational mechanisms or utility to a focus on how those educational mechanisms become contextualized by the individual in a cultural/professional value system.

As a way to stimulate further discussion of the roles of theoretical or conceptual orientations in simulation research, particularly to understand *why* practicing clinicians engage with simulation for CPD, consider as examples what implicit theories of intelligence [46], self-determination theory [47], theory of planned behavior [48] and identity theory [49] may offer. Implicit theories of intelligence for instance highlight how individuals may perceive intelligence, perhaps competence or performance as well, as being either fixed or malleable. This can alter one’s goal orientation along a continuum from performance-based such as seeking opportunities to demonstrate competence to learning-focused involving the seeking of opportunities to reveal gaps and improve. The example provided by Mutaabdzic above highlights how a performance mind-set may be more predominately a cultural/professional value than a growth mind-set. Self-determination theory provides a lens by which to examine the social, psychological, and educational conditions—specifically autonomy, relatedness, and competence—that may foster or antagonize learning or engagement in learning opportunities. The theory of planned behavior similarly emphasizes the role of behavioral beliefs, normative, or subjective beliefs and intention. Finally, identity theory describes how being and doing are central features of identity and that when attempting to understand what defines, shapes, or threatens identity, researchers and educators must focus on the person as well as their role and peer group. An individual clinician’s actions are shaped by how those actions align, advance, or threaten assumed identities. Applied to simulation in a CPD setting, these and other theories may lead to re-thinking or at least seeing differently how we plan for and organize simulations, what we intentionally and unintentionally communicate about them, what we do to shape how they are perceived by individuals and groups and what we might do about fostering more agency and affordances. These theoretical frameworks are provided here as examples only, but common among them are ways of shifting what is studied and what can be learned beyond utility and educational mechanisms to an interaction between those features and the individuals, groups and professional cultures we hope will participate.

An underlying assumption in CPD is that practicing clinicians will select the content and learning methods that best improve their performance toward better clinical outcomes [50]. Even when this is true, simulation has seldom been integrated as a desired or selected CPD strategy. In light of (a) the educational affordances now associated with contemporary healthcare simulation, (b) the pace at which clinical practice evolves, (c) the close relationship practicing clinicians have with healthcare outcomes, (d) evidence that experience is not a surrogate for expertise, and (e) that preparing for autonomy may not adequately meet the needs required to respond to changes once in practice [12, 13], simulation-based research on “why” clinicians engage in simulation or not may thus serve as a renewed “ethical imperative” for the community. To date the research evidence falls short of clarifying whether and why simulation can be successfully integrated as a CPD activity, particularly when extrinsic motivators such as maintenance of certification are removed or absent. A broader set of theoretical perspectives will help us probe the psychological, social and professional cultural factors that dictate the lived experiences of practitioners when deciding on their CPD. Ziv’s claim that “simulation-based education has the potential to decrease the numbers and effects of medical errors … [and] to enhance patient safety” [2] may become *more true* when we understand deeply why those closest to said outcomes engage with and benefit from simulation-based educational activities. The scientific simulation community has a responsibility to make this exploration a “road well traveled.”

## Data Availability

Not applicable

## Abbreviation

CPD: Continuing professional development
